# Beau’s Lines on Fingernails and Toenails

**DOI:** 10.31662/jmaj.2025-0326

**Published:** 2025-11-21

**Authors:** Masato Shimozono, Takafumi Kato

**Affiliations:** 1 Center for Pulmonary Diseases, NHO Tokyo National Hospital, Kiyose, Tokyo, Japan

**Keywords:** Beau’s line, chemotherapy, nail change

A 77-year-old man with metastatic lung adenocarcinoma received 21 cycles of docetaxel chemotherapy at three- to five-week intervals as his third-line regimen after failures of afatinib (first-line) and carboplatin plus pemetrexed plus pembrolizumab (second-line). After docetaxel initiation, multiple transverse lines developed on the patient’s fingernails (Figure 1) and toenails (Figure 2). These nail changes were estimated to appear at the proximal nail fold approximately four to five weeks after each administration. The nail changes were not accompanied by pain or periungual erythema. Mild skin hyperpigmentation, also attributed to docetaxel, was noted on the dorsum of his fingers and toes; however, no other significant skin adverse events were observed.

**Figure 1.**
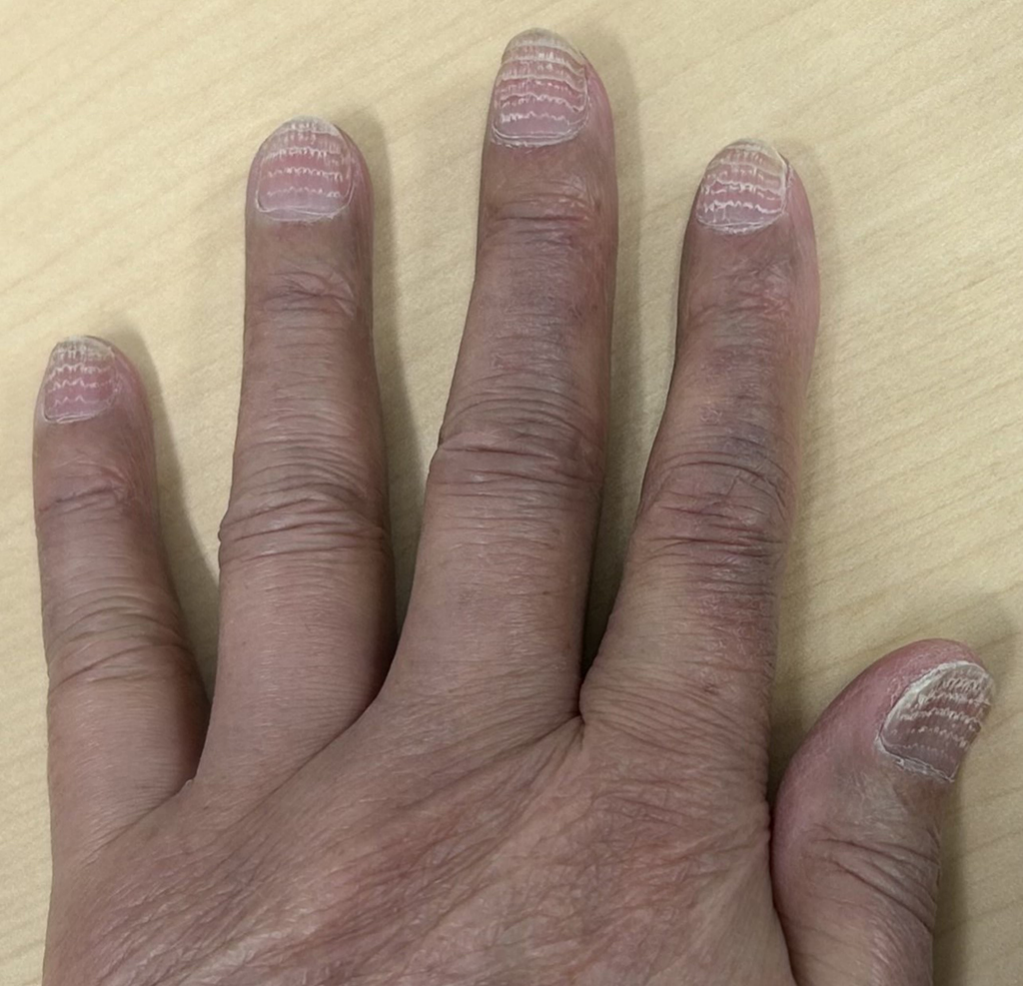
Fingernails with multiple transverse lines.

**Figure 2.**
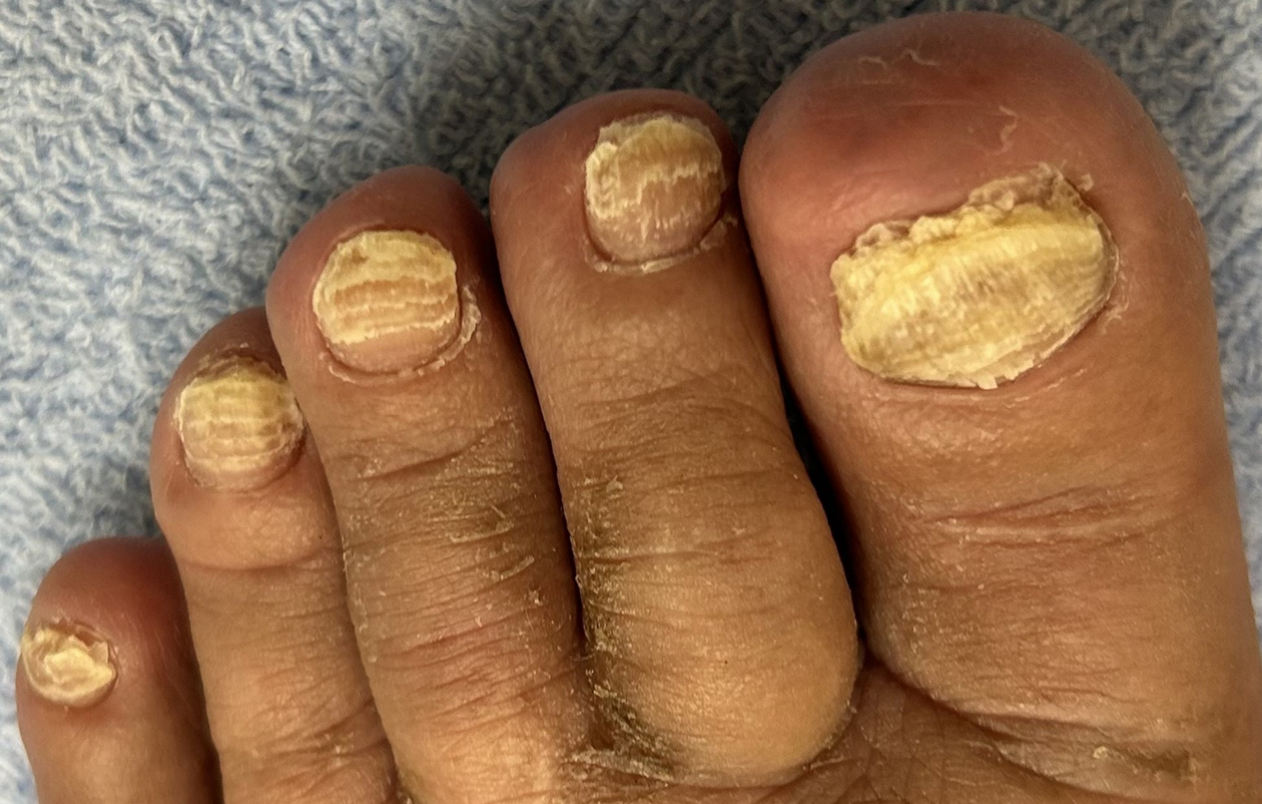
Toenails with multiple transverse lines.

These transverse grooves are known as Beau’s lines, which result from a temporary arrest of nail matrix proliferation. Cytotoxic agents, particularly docetaxel, are well-known causes ^(1), (2)^. In this case, the spacing between the lines was consistent with nail growth over the three- to five-week chemotherapy cycles. Other potential etiologies, such as severe systemic infection, major trauma, or other systemic diseases, were excluded on the basis of the patient’s clinical course. The clear temporal relationship, combined with the observation that line formation ceased after the discontinuation of docetaxel, strongly implicates docetaxel as the causative agent. These depressions should be distinguished from Mees’ lines, which are white, non-palpable transverse bands resulting from abnormal keratinization of the distal nail matrix, also associated with chemotherapy ^(1), (2)^.

## Article Information

### Author Contributions

Masato Shimozono and Takafumi Kato were responsible for patient management, and manuscript writing and review.

### Conflicts of Interest

None

### IRB Approval

Not required for a case report/image.

### Patient’s Consent

Written informed consent was obtained from the patient for the submission and publication of this case report/image.

## References

[1] Piraccini BM, Iorizzo M, Tosti A. Drug-induced nail abnormalities. Am J Clin Dermatol. 2003;4(1):31-7. 12477371 10.2165/00128071-200304010-00004

[2] Kinjo T, Shibahara D, Higa F, et al. Beau’s Lines and Mees’ lines formations after chemotherapy. Intern Med. 2015;54(17):2281. 26328664 10.2169/internalmedicine.54.5166

